# Hydrogels as Durable Anti-Icing Coatings Inhibit and Delay Ice Nucleation

**DOI:** 10.3390/molecules25153378

**Published:** 2020-07-25

**Authors:** Beili Huang, Shanshan Jiang, Yunhe Diao, Xuying Liu, Wentao Liu, Jinzhou Chen, Huige Yang

**Affiliations:** School of Materials Science and Engineering, Zhengzhou University, Zhengzhou 450001, Henan, China; hbl@gs.zzu.edu.cn (B.H.); jiangshans@gs.zzu.edu.cn (S.J.); yunhediao@gs.zzu.edu.cn (Y.D.); liuxy@zzu.edu.cn (X.L.); wtliu@zzu.edu.cn (W.L.); cjz@zzu.edu.cn (J.C.)

**Keywords:** anti-icing coating, hydrogels, cross-linking, sodium alginate

## Abstract

The accumulation of ice on surfaces brings dangerous and costly problems to our daily life. Thus, it would be desirable to design anti-icing coatings for various surfaces. We report a durable anti-icing coating based on mussel-inspired chemistry, which is enabled via fabricating a liquid water layer, achieved by modifying solid substrates with the highly water absorbing property of sodium alginate. Dopamine, the main component of the mussel adhesive protein, is introduced to anchor the sodium alginate in order to render the coating applicable to all types of solid surfaces. Simultaneously, it serves as the cross-linking agent for sodium alginate; thus, the cross-linking degree of the coatings could be easily varied. The non-freezable and freezable water in the coatings with different cross-link degrees all remain liquid-like at subzero conditions and synergistically fulfill the aim of decreasing the temperature of ice nucleation. These anti-icing coatings display excellent stability even under harsh conditions. Furthermore, these coatings can be applied to almost all types of solid surfaces and have great promise in practical applications.

## 1. Introduction

Ice formation and accumulation on surfaces causes serious problems in our daily life, including the difficulty of operation and high maintenance efforts of ground transportation vehicles and power networks [1,2,3,4,5]. In some cases, ice on surfaces even causes disastrous events; for example, 12% of aircraft crashes are attributed to icing on the wings resulting in great losses of life and property [6]. Over the past few decades, scientists have proposed two main strategies, active methods and passive methods, for fighting against icing. The active methods refer to the removal of the accumulated ice, which usually is costly and time-consuming [7,8,9,10,11,12,13]. Therefore, it is much more desirable to design passive anti-icing surfaces via lowering freezing temperature or reducing ice adhesion, with the benefit of reducing energy consumption [8]. Recently, several strategies have been proposed to depress the freezing temperature, such as ion-infused surfaces [14,15], charged surfaces [16], photothermal surfaces [17], etc. Meanwhile, Wang and co-workers found that water molecules can interact tightly with hydrophilic polymers, making the water in the hydrogel coating remain liquid in subzero environments, a quality which can be utilized to reduce the ice adhesion. Dayong et al. [18] also discovered that the water in the polydimethylsiloxane (PDMS)-poly(ethylene glycol) (PEG) (PDMS-PEO )hybrid coating can also reduce ice adhesion because of the tight bonding between water molecules and PEO. These kinds of aqueous lubricating coatings do not have the problem of durability because water can be resupplied from moisture or melted ice, making them ideal anti-icing coatings [19,20,21,22,23,24,25,26]. However, there are some key questions that remain unanswered. For example, how much can the temperature be lowered by different hydrophilic polymers? How does the cross-linking degree affect the freezing temperatures?

Here, we investigated the effects on the freezing temperature of hydrogels. Hydrogels are cross-linked hydrophilic polymers, swollen by large amounts of water, which have wide applications for tissue engineering and drug delivery and cryopreservation [27,28,29]. Inspired by adhesive proteins in mussels [30,31], the highly hydrophilic polysaccharide sodium alginate (SA) is attached onto substrates, forming hydrogel coatings. Sodium alginate is a natural linear polysaccharide which has been applied as various types of biocompatible hydrogels [32,33]. The strong association of water molecules with the SA chain can depress the freezing temperature greatly, leading to a layer of unfreezing water that remains liquid-like down to low temperatures, rather than freezing upon encountering humid conditions [34]. Compared with the traditional materials of anti-icing coatings that function by constructing superhydrophobic surfaces or maintaining a lubricating layer of oil infused into micro/nanostructured substrates [10,20], which is susceptible to malfunction due to various possibilities, hydrogels retain their potentiality as long as water is present and thus maintain their long-lasting anti-icing properties. Specifically, SA is firstly conjugated with catechol via the reaction of SA with dopamine hydrochloride (DA); secondly, the conjugation SA-g-DA is deposited onto a variety of substrates in a substrate-independent fashion; thirdly, sodium periodate (NaIO_4_) is added for oxidative cross-linking, constructing hydrogel surfaces with different cross-linking densities. The results demonstrate the ability of hydrogel coatings to lower freezing temperatures. Meanwhile, the catechol-conjugated SA can be modified onto various surfaces because catechol can adhere to different substrates. In addition, the addition of sodium periodate maintains excellent stability and avoids the chemical pretreatment of substrates. These features in turn endow the anti-icing hydrogel coatings with universality and stability.

## 2. Results

### 2.1. Synthesis of and Characterizations of the SA-g-DA

**Figure 1:**
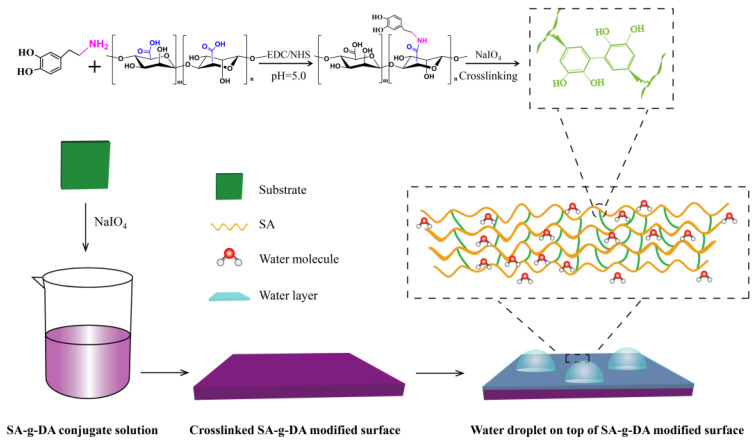
Schematic representation of the process of the durable anti-icing coating.

**Figure 2:**
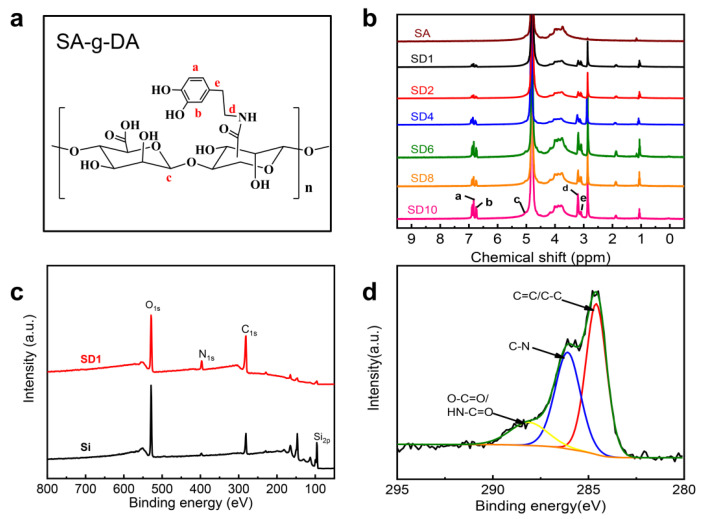
(**a**) Structure of SA-g-DA conjugate(the reaction product of sodium alginate and dopamine); (**b**) ^1^H-NMR spectra of SA and SA-g-DA conjugate; (**c**) X-ray photoelectron spectra of the silicon wafer surface (bottom) and the surface coated by SD1 conjugate and cross-linked via NaIO_4_ (top), where SD1 denotes the silicon wafers functionalized with SD1 conjugate; (**d**) high-resolution XPS spectra of C 1s.

In this work, SA-g-DA was synthesized by the conjugation of DA with SA via carbodiimide coupling chemistry (1-(3-dimethylaminopropyl)-3-ethylcarbodiimide hydrochloride/N-hydroxysu- ccinimide, EDC/NHS), as shown in Figure 1. The chemical composition of the conjugation SA-g-DA was analyzed by UV-Vis, FTIR and ^1^H-NMR spectrum spectroscopy. As shown in Figure 2b, the peak of ^1^H-NMR at around 7 ppm in the SA-g-DA conjugate is the chemical shift of aromatic protons on DA phenyl (marked a and b in Figure 2a). After grafting DA, two new peaks (labeled d and e) appeared on the N=CH_2_ and C-CH_2_ protons at d = 3.25 and e = 3.15, respectively [28,33]. A peak at 280 nm, typical of the catechol structure of the SA-g-DA solution in ultraviolet–visible spectroscopy [21], and the amide bond (1717 cm^−1^) in Fourier transform infrared (FT-IR) spectroscopy [28] also support this result (Figure S1). All the results demonstrated that the SA has been introduced into DA.

To calculate the grafting ratio (f), we assume that SA is constructed from the main sequence, and each repeating unit (Mw = 198 Da) has one sodium carboxylate and several sodium sulfonate groups. Therefore, the grafted carboxyl group can be calculated from the relative peak intensity ratio of the anomeric hydrogen proton c in SA (5.00–5.30 ppm) to the aromatic protons (a and b) in DA (6.70–7.40 ppm) [29]. As the amount of DA increases, the f increases accordingly. The calculated graft ratios from the ^1^H-NMR spectra are shown in Figure S2 (Table 1). In humid conditions, the cross-linked network of SA-g-DA conjugates will swell to a certain extent to open the network after hydration, allowing more water molecules to penetrate into the network until the maximum water absorption (MWU) is reached. However, as the grafting rate f increases, the swelling ratio is reduced, resulting in the values of MWU decreasing from 32.4% to 5.0% (Figure S2 and Table 1).

### 2.2. Synthesis of the Durable Anti-Icing Coating

Figure 1 illustrates the fabrication procedure of the durable anti-icing coating. The SA-g-DA conjugates are deposited onto a variety of substrates by mussel-inspired surface chemistry. Then, sodium periodate (NaIO_4_) is added for oxidative cross-linking. Catechol is used as an anchoring group to deposit the conjugate onto the substrate simultaneously with the site to cross-link the conjugate via NaIO_4_-induced dismutation for the purpose of improving the durability of the coating. The cross-linking degree of the coatings can be tailored via changing the molar feed ratio of SA to DA (Table 1) to investigate the effect of cross-linking degree on the ice nucleation. The role of cross-linking played by NaIO_4_ can be corroborated by performing NaIO_4_-mediated oxidation of a solution of SA-g-DA conjugate (Figure S3).

**Figure 3:**
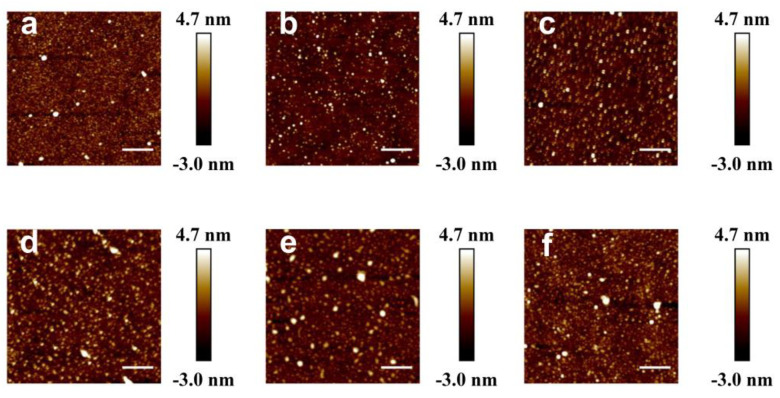
Atomic force microscope (AFM) height images (2μm × 2μm) of hydrogel coating (SD1) deposited on silicon wafers before (**a**) and after (**b**) cross-linking in the air, (**c**) SD8 coating, immersed in (**d**) 0.1 M NaOH, (**e**) 0.1 M HCl and (**f**) 5 M NaCl for 2 h.

Silicon wafer is selected as a model substrate to prepare the hydrogel surfaces. X-ray photoelectron spectroscopy (XPS) is utilized to characterize the variations of surface elements, verifying the formation of the coating and the cross-linking between SA−g-DA conjugates. The XPS spectra of silicon surfaces before and after modifying SD1 are shown in Figure 2c. The surface functionalized by SD1 shows the emergence of a new peak of *N* 1s and a decrease in the intensity of the Si 2p peak compared with the bare silicon wafer, which means that SD1 was successfully coated on the silicon wafer surface. The XPS C1s spectra of the SD1 surface are shown in Figure 2d; the binding energy at 286.0 eV was attributed to C–N originating from the amide bond, and the signal at around 288.1 eV was attributed to C=O, corresponding to the carbonyl carbon of SA-g-DA. Typically, the SD1 O1s spectrum shows three unique nitrogen moieties, C=O at 531.5 eV, O–C=O at 532.3 eV and C–O at 533.0 eV, which are consistent with the above C1s. There are two components of the N 1s peak, corresponding to the amine groups (R-NH_2_, 401.5 eV) and substituted amines (R-NH-R, 399.8 eV), indicating the cross-linking between SA−DA conjugates [21,28,35]. These results further confirmed that SA-g-DA was successfully adhered onto the silicon wafer surfaces (Figure S4). Dopamine hydrochloride (DOPA) performs well as a binding agent by the coordination bonds and π–π interaction [36]. Thus, dopamine modified with SA-g-DA conjunction can also be coated onto all types of surfaces in a substrate-independent fashion. Afterwards, the water contact angle provides a way to investigate the effect of SA-g-DA coatings on the surface properties. We chose five samples for each cross-linking degree; no less than three different measurement points was selected from each sample and took the average value as the value of the contact angle. After coating SA-g-DA, the contact angles of a series of surfaces from SD1 to SD10 fluctuated between 38° and 62.2° due to the different swelling rates of the hydrogel surfaces caused by the amount of cross-linking agent [33]. The variation in surface roughness (Figure 3b,c), the value of contact angle (Figure S5) and surface chemistry characterized by X-ray photoelectron spectroscopy (XPS) (Figure 2c,d) confirmed that the SA-g-DA was successfully coated onto the surfaces.

### 2.3. Stability of the Anti-Icing Coating

The morphology of coatings was investigated by an atomic force microscope (AFM). Images a and b in Figure 3 indicate that the coating of SD1 seemingly has an indistinguishable effect on surface morphology; the average surface roughness (*R*_a_) of cross-linked and un-cross-linked coatings is 0.71 nm and 0.47 nm, respectively. Images b and c in Figure 3 represent the AFM images of the SD1 and SD8 coatings, respectively. The negligible variation in morphologies before and after cross-linking demonstrates the gentleness characteristic of catechol chemistry, which is superior to thermally induced cross-linking. To further probe the stability of this coating, we conducted soak testing in three types of etching solutions. As shown in Figure 3d,f (Table S1), the SD1 coating still maintains its morphological integrity after treatment with the solutions. Moreover, the freezing temperatures (T_h_) of water droplets on the SD1 coated surface (T_h_ (SD1) = −23.8 ± 1.0 °C) remain almost unchanged after soaking in acid, alkaline and salt solution (T_h_ (NaCl) = −24.2 ± 1.0 °C, T_h_ (NaOH) = −24.0 ± 1.2 °C, T_h_ (HCl) = −23.6 ± 1.0 °C), illustrating the outstanding stability of the cross-linked hydrogel coatings.

### 2.4. Anti-Icing Performances

**Figure 4:**
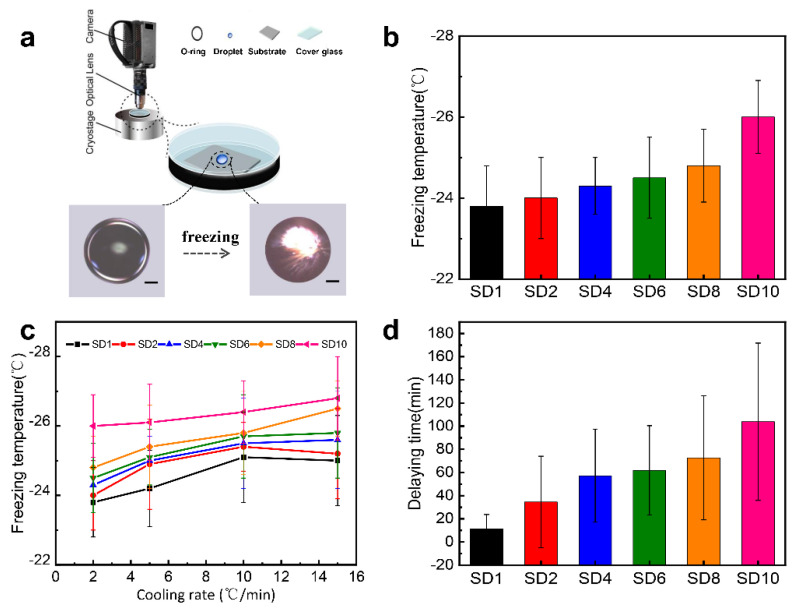
(**a**) Microscopic images of water macrodroplets; (**b**) ice nucleation temperatures on a series of substrates coated by the cross-linked SA−DA conjugates (SD1 to SD10) during cooling from ambient temperature at a rate of 2 °C min^−1^; (**c**) the dependence of T_h_ upon the cooling rate; (**d**) average delay times of ice nucleation measured at –23 °C on the hydrogel surfaces with different cross-linking degrees.

The ice nucleation temperature was performed by a homebuilt closed cell, as described elsewhere [16,37,38]. Specifically, three to four equally-sized macrodroplets of 0.1 μL pure water droplets (18.2 MΩ cm) were independently placed on the hydrogel surfaces (from SD1 to SD10) so as to avoid freezing by triggering/inducing with the side water droplets as much possible. Then, the closed cell was placed atop a cryostage (Linkam THMS 600, Tadworth, UK) to cool down. Water freezing is a process of ice crystallization from supercooled water. In this process, water undergoes two stages, i.e., ice nucleation and growth of ice crystals. As compared with the slower ice nucleation, the rapid growth leads to the instant freezing of water microdroplets upon the formation of ice nuclei. Thus, the sudden change in opacity before and after freezing observed can be identified as ice nucleation (Figure 4a). Since the volume of the closed cell was small enough, the water droplet thermodynamically equilibrated with the water vapor in the closed cell. In such an experimental condition, the relative humidity was 100%. In addition, the entirety of the sample cell preparation was conducted in a clean room. To acquire the ice nucleation temperature, we lowered the surface temperature and recorded the temperature when the droplet became opaque. Since the cooling rate (2 °C min^−1^) is relatively low, it is reasonable to state that the water droplet is in a quasi-static equilibrium with the water vapor when the surface temperature is slowly lowered. Then, we have carried out a “single droplet ice nucleation experiment” by repeating “cooling down-ice formation and then heating up-ice melting” at least three times, with which we obtained the average ice nucleation temperature on each individual hydrogel surfaces. Only through this process can we obtain reliable ice nucleation temperatures. The criterion that we obeyed in judging whether the ice nucleation events were adequate was whether the dataset could be fitted with the Gaussian function to give an average nucleation temperature. In this work, we found that the number of freezing temperature histories for 100 freezing events gave a good fit by the Gaussian function (Figure S6).

As shown in Figure 4b, the ice nucleation temperature (T_h_) on the SD1 surface is −23.8 ± 1.0 °C, with a cooling rate of 2 °C min^−1^, whereas it is −26.0 ± 0.9 °C on the SD10 surface. The T_h_ on the hydrogel surfaces with different cross-linking are all below −23 °C and can be switched by changing the molar ratio of SA to DA (Table 1). Additionally, the increase in f gradually lowers the T_h_ in the sequence of SD1 > SD2 > SD4 > SD6 > SD8 > SD10. In order to explore the effect of the cooling rate on the freezing temperature of the hydrogel surfaces with different cross-linking degrees, we used the cooling rates of 2 °C min^−1^, 5 °C min^−1^, 10 °C min^−1^ and 15 °C min^−1^ to measure the nucleation temperature of water droplets on a series of hydrogel surfaces, as shown in Figure 4c. The results show that, when the cooling rate is changed, the ice nucleation temperature trend of the hydrogel surfaces with different cross-linking is consistent—that is, as the cross-linking degree increases, the freezing temperature of water droplets gradually decreases.

The anti-icing performances of coatings were consolidated by the delay time assay of ice nucleation, which was measured by maintaining a specific supercooling temperature at −23 °C and then recording the time needed for ice nucleation to occur. Ice nucleation occurred on the SD1 surface at 10 min, and after a delay time of 100 min, ice nucleation occurred on the surface modified with SD10, with a relative humidity of 100% in the closed chamber. We found again that the delay time of ice nucleation was also consistent with the order of nucleation temperature: t_d_ (SD1) < t_d_ (SD2) < t_d_ (SD4) < t_d_ (SD6) < t_d_ (SD8) < t_d_ (SD10) (Figure 4d). The sequence of the delay time agrees well with the freezing temperature obtained from the above results, consolidating the experimental results.

**Figure 5:**
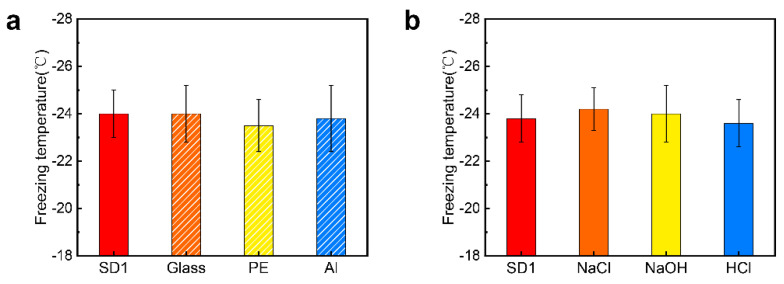
(**a**) Comparison of the ice nucleation temperature measured at 2 °C min^−1^ on a wide range of substrates after the application of the hydrogel coating (SD1). Al and PE denote aluminum and polyethylene, respectively. (**b**) The value of ice nucleation temperature on the hydrogel coating (SD1) after immersion into three types of solution including alkaline, acid and salt solutions.

To further consolidate the versatility of the anti-icing coating presented by the hydrogel, we used the same method to adhere the SD1 coating to virtually all types of solid surfaces, including glass, polyethylene and aluminum. The results demonstrate that our anti-icing hydrogel coatings have the following advantages: 1) this anti-icing coating can be easily constructed by immersing all kinds of substrates into the SA-g-DA solution; 2) the anti-icing coatings can be fabricated at room temperature and do not require any chemical pretreatment of substrates; 3) the cross-linking density of the coatings would be varied due to the covalent oxidative coupling of dopamine, which is crucial for tailoring the anti-icing performances. Moreover, the ice nucleation temperatures for the cross-linked coatings after soaking in the solutions indistinguishably vary, including the HCl, NaOH and NaCl solutions, and are almost the same as those of their counterparts without soaking. As shown in Figure 5b, T_h_ (NaCl) = −24.2 ± 1.0 °C, T_h_ (NaOH) = −24.0 ± 1.2 °C, T_h_ (HCl) = −23.6 ± 1.0 °C and T_h_ (SD1) = −23.8 ± 1.0 °C. These results illustrate that the stability of the coating would be enhanced through NaIO_4_-induced cross-linking, and the anti-icing performances of cross-linked coatings remain existent even in a harsh environment, which is consistent with the literature [39].

## 3. Discussion

It has been reported that water in the hydrophilic polymer exists as two states, i.e., non-freezable water, which interacts with the hydrophilic functional groups, such as carboxyl groups on polymer chains, to prevent this water from freezing, and freezable water existing in the macromolecular interstices and/or with proximal occupancy around the attached water molecules [23,40,41,42]. Damrongsak et al. [34] used differential scanning calorimeter (DSC) to measure the distribution of water in sodium alginate and found that there were two exothermic peaks during the cooling process, which were the crystallization of conjugated bound water (non-freezable water) at −23 °C and crystallization of free water at −16 °C, respectively, where the large decrease in the crystallization temperature of the freezable bound water is due to the polymer–water interaction. In our work, we added three to four drops of 0.1 μL ultrapure water droplets onto the surface of the hydrogel to test the ice nucleation temperature. Since sodium alginate is a highly water-absorbing hydrophilic polymer, which quickly absorbs water during the cooling process, some non-freezable water forms a hydrogen bond with the polar functional groups on the sodium alginate hydrogel (locally favorable configuration). This is strong enough to prevent the water from freezing. The average ice nucleation temperature of the water droplets on the hydrogel surface with different cross-linking degrees observed in our experiments is below −23 °C, which is close to the position of the exothermic peak of the crystallizable bound water. Therefore, we speculate that there is freezable-bound water in the hydrogel.

Okoroafor et al. [34] have reported that, under fully hydrated conditions, the amount of non-freezable water (C_unf_) increases with the increase in cross-linking degree, and the increase in cross-linking degree leads to a reduction in the amount of freezable water (C_f_). As described later, both the non-freezable water and the freezable water remain liquid in the coatings under subzero conditions and work synergistically to reduce the temperature of ice nucleation. The increase in f increases the proportion of C_unf_ in the network from SD1 to SD10, enabling non-freezable water with a lower freezing point. At the same time, as f increases from SD1 to SD10, C_f_ gradually decreases, but the freezing temperature gradually decreases from SD1 to SD10, as described below. Thus, the synergistic effect between the none-freezable and freezable water lowers gradually the ice nucleation temperature from SD1to SD10. Meanwhile, the increase in cross-linking degree up to a critical degree significantly reduces MWU to such an extent that the network cannot possess sufficient water for the liquid water layer. Therefore, the ice nucleation temperature on the surface of SD10 reaches the lowest value.

To quantify our results, it is assumed that only the freezable water freezes when the temperature is cooled to its freezing point. At the same time, the non-freezable water combines with the gel to form the gel phase of the pore wall after polymerization. When the temperature is lowered to freezing temperature T_c_, the equilibrium condition between the ice phase ($\mu_{1}^{ice}$) and the gel phase ($\mu_{1}^{gel}$) is that the chemical potentials of the two are equal, i.e., $\mu_{1}^{ice}=\mu_{1}^{gel}$, in the gel phase $\mu_{1}^{gel}=\ln a_{n}-RT\ln a_{A}$, where $\mu_{1}^{0}$, $a_{A}$ and are the chemical potential of pure liquid water and the activity of water in the gel, respectively. When the ice melts, $\mu_{1}^{ice}=\mu_{1}^{gel}$ equal to the change in the molar Gibbs free energy. One obtains [43] the following:

(1) $$\ln a_{A}=\frac{\Delta H_{m}}{R}\left(\frac{1}{T_{0}}-\frac{1}{T_{c}}\right)$$

where $\Delta H_{m}$ is the molar enthalpy of melting ice, $T_{0}$ is the normal freezing point of pure water, and *R* is the gas constant. The Flory theory (including Donnan equilibria) had given the following relation in the solvent–polymer network between the solvent’s crystallization temperature and the gel parameters:

(2) lnaA=ln(1−φ2)+φ+χ1φ22+V1(υeV0)(φ213−φ22)−fφ2

where $\varphi_{2}$,$\chi_{1}$,$\upsilon_{e}$ and f are the volume fraction of the gel polymer in the hydrogel network, the polymer–solvent interaction parameter, the effective cross-link density in the polymer network and the effective charge density, respectively. $V_{1}$ and $\varphi_{2}$ are the molar volume of solvent and the volume fraction of polymer network in the gel, the freezing temperature $T_{c}$ of water confined in a network of polymer chains as a function of the network parameters [39]. Finally, $\upsilon_{e}$ is the effective cross-link density of the network, substituting Equation (2) into Equation (1):

(3) (1Tc−1T0)ΔHmR=−ln(1−φ2)−φ2−χ1φ22−V1(υeV0)(φ213−φ22)+fφ2

The first three terms on the right in Equation (3) represent the chemical potential of a general polymer–water mixture; the fourth term is the chemical potential due to the cross-linked network structure, and the last term is derived from Donnan equilibria theory. In this study, sodium alginate hydrogels with different cross-linking degrees were used. The consistency of the material can exclude the influence of charge, so Equation (3) can be simplified to [39]:

(4) (1Tc−1T0)ΔHmR=[−ln(1−φ2)−φ2−χ1φ22]+[ρe(φ22−φ213)]

where $\rho_{e}=V_{1}(\upsilon_{e}/V_{0})$ explains the influence of the cross-linked structure and can be deduced from Equation (4): for the freezing temperature $T_{c}$ of the unfreezing water in the hydrogel, an increase in $\varphi_{2}$ causes a decrease in $T_{c}$ for the first item in parentheses and a tendency to increase $T_{c}$ for the second item in parentheses, which is related to the crystallization process The medium expansion network has a tendency to drain. However, since the value of $\rho_{e}$ that is usually encountered is 0.01–0.02, the influence of the second term is quite small, so that the increase of $\varphi_{2}$ causes the decrease of $T_{c}$.

Chen et al. [39] has reported that the freezing temperature $T_{c}$ of the freezable water in the hydrophilic polymer and the water volume fraction $\varphi_{2}$ in the hydrogel network have a functional relationship when $\chi_{1}=0.50$. As shown in Figure S7, the water remains liquid until it is below zero; as the water content of the hydrogel decreases, the freezing temperature gradually decreases, and the downward trend gradually increases. In our work, the content of the non-freezable water and freezable water changed in the hydrogel with the increase in the cross-linking degree, simultaneously causing the decrease in freezing temperature. This conclusion is in agreement with the reports presented in the literature [18].

## 4. Materials and Methods

### 4.1. Materials

Sodium alginate (SA, Mw = 96000 Da, Macklin, Shanghai, China), NHS (99%, Alfa Aesar, Shanghai, China), EDC (99%, Alfa Aesar), dopamine hydrochloride (99%, Alfa Aesar), dialysis membranes (molecular weight cutoff (MWCO) = 3500, Viskase, Shanghai, China) and sodium periodate (NaIO_4_; 99%, Macklin) were used as received.

### 4.2. Synthesis of SA-g-DA Conjugate

A total of 30 mL phosphate buffered saline (PBS) was prepared in advance, adjusting to pH = 5 with hydrochloric acid (1 mol L^−1^); then, 89.1 mg of sodium alginate (SA) was dissolved in PBS buffer, stirring for 30 min; then, 26.226 mg of EDC and 43.68 mg of NHS were added to the mixture to activate the carboxylic group of hyaluronic acid; then, a certain proportion of DA (0 mg, 17.067 mg, 34.134 mg, 68.268 mg, 102.402 mg, 136.536 mg, 170.67 mg) was added to the SA solution for 9 h, with continuous stirring at room temperature, respectively. Finally, the solution was dialyzed transferred by a dialysis membrane (MWCO = 3500) in deionized water for 24 h to such an extent that the DA in the washing solution was not tested by UV-Vis spectroscopy. The powder sample was obtained by freeze-drying of the above solution, and it was sealed for use and named SA, SD1, SD2, SD4, SD6, SD8, SD10.

### 4.3. Fabrication of SA Hydrogel Surface

Silicon wafers (7 mm × 7 mm), polyethylene, aluminum, glass and cover glasses (Ø = 22 mm) were sequentially cleaned by sonication in ethanol, acetone and ultrapure water. The conjunction of SA-g-DA was dissolved in PBS solution at a concentration of 10% (*w*/*v*). Then, equimolar sodium periodate (NaIO_4_) was added to cross-link the conjugate. The substrates were immersed in the above solution for 6 h; then, we removed the substrates and dried them with high purity nitrogen.

### 4.4. Characterization

The conjugation of SA with DA was analyzed by Fourier transform infrared (FT–IR) spectroscopy (BRUKER TENSOR II, Bremen, Germany). The UV-Vis spectra were acquired by a UV-Vis spectrophotometer (Agilent Cary 3500, California, Malaysia). The chemical compositions of the hydrogel surfaces were characterized with XPS (AXIS Supra, Manchester, England). The measurement of static water contact angles (CAs) on the sample was conducted with CA System (JC2000C1, Shanghai, China). The AFM images were acquired from Dimension Icon (Bruker, Bremen, Germany). ^1^H-NMR (400 MHz) spectra were recorded using a NMR Spectrometer (Bruker, Karlsruhe, Switzerland).

### 4.5. Fabrication of Durable Hydrogel Coatings

Inspired by the composition of adhesive proteins in mussels, using the surface chemistry to prepare anti-icing coatings has been employed as a simple and universal strategy. The SA is firstly conjugated with the catechol via the reaction of SA with dopamine hydrochloride; secondly, the conjugation SA-g-DA is deposited onto a variety of substrates in a substrate-independent fashion; thirdly, sodium periodate (NaIO_4_) is added for oxidative cross-linking, constructing the hydrogel surfaces with different cross-linking densities.

### 4.6. Measurement of Ice Nucleation Temperature and Delay Time

The entire experiment was carried out in a clean room. The measurement of the temperature and delay time with ice nucleation on the SA-g-DA surface was operated in a tailor-made chamber, as described elsewhere [16]. Subsequently, three uniformly sized macrodroplets of 0.1 μL pure water droplets (18.2 MΩ cm) were independently placed on the hydrogel surface (from SD1 to SD10) so as to avoid freezing as much possible by triggering/inducing with the side water droplets [44]. Then, the closed chamber was placed atop a cryostage (Linkam THMS 600, Tadworth, UK) to cool down at different cooling rates, namely 2 ° C min^−1^, 5 °C min^−1^, 10 °C min^−1^ and 15 °C min^−1^, to further study the effect of cross-linking degree on ice nucleation temperature. In order to acquire reliable statistics, each freezing experiment was repeated 100 times to record the temperature of ice nucleation.

Meanwhile, the delay time of ice nucleation was measured also in the same operation. A macrodroplet of 0.1 μL ultrapure water (18.2 MΩ cm) was placed on the hydrogels’ surfaces to be tested. Firstly, the substrate was cooled to a targeted temperature (−23 °C) and maintained constantly until it freezes. The delay time of ice nucleation (t_d_) was defined as the difference between the starting time when the substrate was cooling to a targeted temperature and the ending time at the moment of a sudden decrease in opacity.

### 4.7. Stability of the Hydrogel Surface

To study the stability of the hydrogels’ surfaces, the silicon wafers modified with SD1 were immersed in HCl, NaOH and NaCl solutions with concentrations of 0.1 M, 0.1 M and 5 M, respectively. After 2 h, they were taken out, rinsed with ultrapure water and dried with high purity nitrogen. The changes in the morphology and anti-icing performance of the solid substrates were measured by using an atomic force microscope and cryostage, respectively.

## 5. Conclusions

In summary, we have developed a new strategy of developing anti-icing coatings which can inhibit and delay ice nucleation. The properties of anti-icing coatings can be maintained by changing the cross-linking degree of hydrogels. We propose that both the non-freezable and freezable water in the coatings with different cross-linking degrees all remain liquid-like at subzero conditions and synergistically fulfill the aim of decreasing the temperature of ice nucleation. The controllability of the cross-linking grants the hydrogel coatings high performance in inhibiting ice nucleation (ice nucleation temperature lower than −23 °C) or delaying ice nucleation (freezing delay time more than 100 min at −23 °C). The subsequent application tests show that anti-icing coatings display excellent stability after cross-linking the SA-g-DA conjugate, even under harsh conditions. Furthermore, the broad applicability to almost all types of solid surfaces will give more opportunities and possibilities for these hydrogels to be a promising candidate for anti-icing coatings. Moreover, the exploration of hydrophilic polymers would provide a simple method to design anti-icing coatings in the future.

## Figures and Tables

**Table 1:** Grafting ratio of SA-g-DA conjugate and water content of the dry SA-g-DA conjugate.

Conjugate	SA (mmol)	DA (mmol)	SA-DA ^a^	f ^b^	MWU
SD1	4.5	0.45	10:1	50.4%	32.4%
SD2	4.5	0.9	10:2	58.8%	21.9%
SD4	4.5	1.8	10:4	59.1%	16.3%
SD6	4.5	2.7	10:6	60.6%	10.1%
SD8	4.5	3.6	10:8	61.3%	6.1%
SD10	4.5	4.5	10:10	83.3%	5.0%

^a^: Molar feed ratio between SA and DA. ^b^ f is the grafting ratio of SA-g-DA calculated from ^1^H NMR spectra. Maximum water absorption (MWU) is determined from weighing method.

## Supplementary Materials

The following are available online: Figure S1: Spectra of SA and SA-g-DA conjugates; Figure S2: The grafting ratio and maximum water uptake of SA-g-DA conjugates; Figure S3: The images of SA-g-DA conjugate solution before and after oxidation by NaIO_4_; Figure S4: High-resolution XPS spectra of O 1s and N 1s of the surface for SD1; Figure S5: Static water contact angles of hydrogel surfaces with different cross-linking degrees; Figure S6: Investigation of the freezing of single macroscopic droplet sitting on hydrogel surfaces (SD1); Figure S7: The freezing temperature of freezable water in the cross-linking system predicted by Flory relation for χ= 0.50; Table S1: The values of average surface roughness, R_a_, of the surface under different condition.
