# Supplementary material for: Hydrogels as Durable Anti-Icing Coatings Inhibit and Delay Ice Nucleation
Source: Molecules. 2020 Jul 25;25(15):3378. doi: 10.3390/molecules25153378 (PMC7435966; doi:10.3390/molecules25153378)
Supplement: Supplementary file 1 [file molecules-25-03378-s001.pdf]

Supplementary Information

Hydrogels as Durable Anti-Icing Coating Inhibit and Delay Ice Nucleation

Beili Huang [1], Shanshan Jiang [1], Yunhe Diao [1] , Xuying Liu [1], Wentao Liu [1], Jinzhou Chen [1],and Huige Yang [1,*]

* Correspondence: yanghg@zzu.edu.cn; Tel.: +86-3716-778-1590

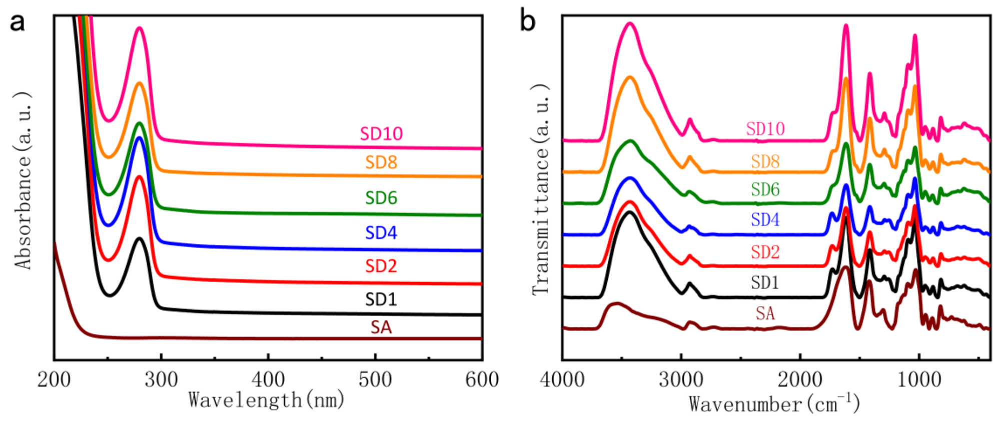

Figure S1. Spectra of SA and SA-g-DA conjugates: (a) UV-vis spectrum; (b) FT-IR spectrum.

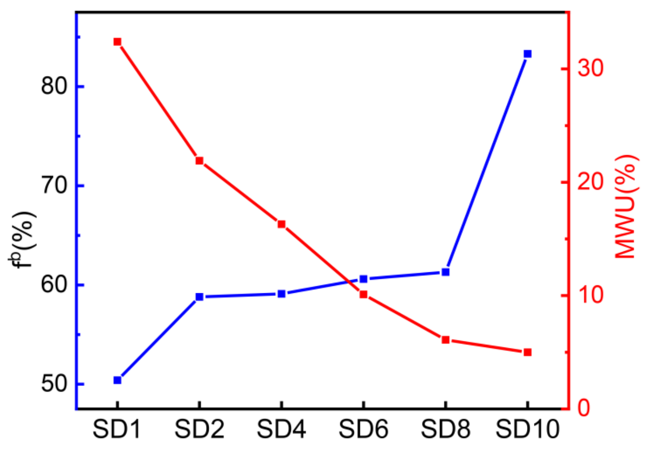

Figure S2. (red) Degrees of substitutions per polysaccharides in the SA-g-DA conjunction, (blue) the maximum water uptake (MWU) of dry SA-g-DA conjugate.

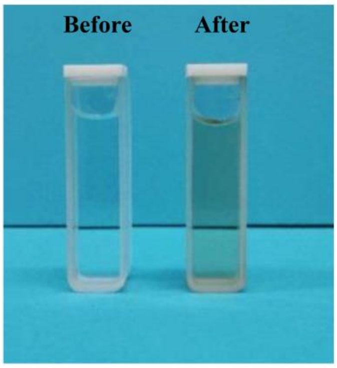

Figure S3. The images of SA-g-DA conjugate solution before and after the oxidation by $NaIO_4$ in the quartz cuvette.

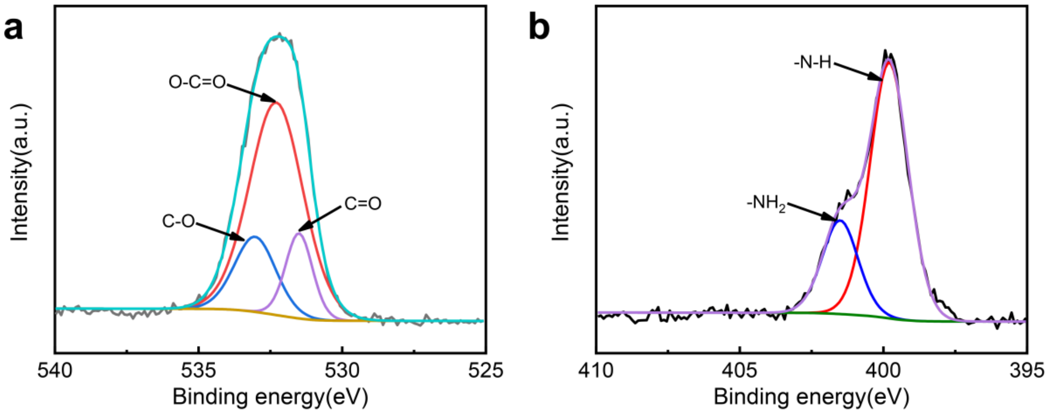

Figure S4. High-resolution XPS spectra of (a) O 1s and (b) N 1s of the surface for SD1.

Table S1. The values of average surface roughness, R_a, of the surface under different condition.

Sample	R_a (nm)
SA-g-DA	0.706
SD1	0.464
SD8	0.718
$SD1_{(NaOH)}$	1.48
$SD1_{(HCl)}$	1.12
$SD1_{(NaCl)}$	0.747

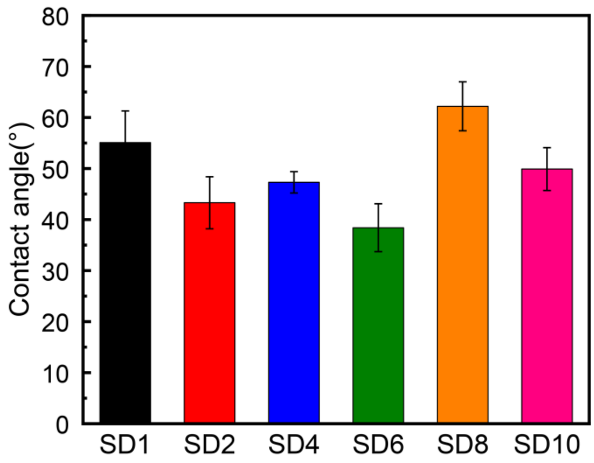

Figure S5. Static water contact angles of hydrogels surfaces with different cross-linking.

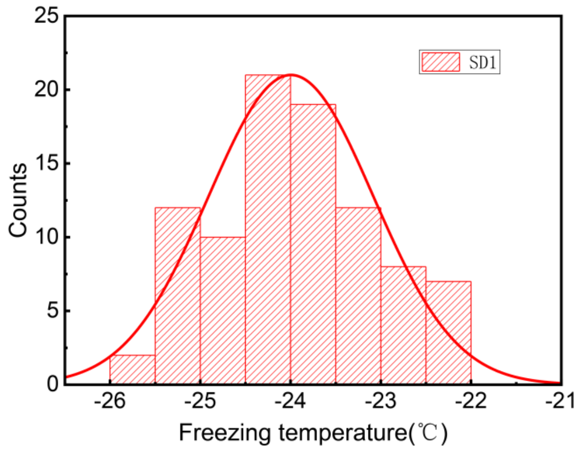

Figure S6. Investigation of the freezing of single macroscopic droplet sitting on hydrogel surfaces (SD1).

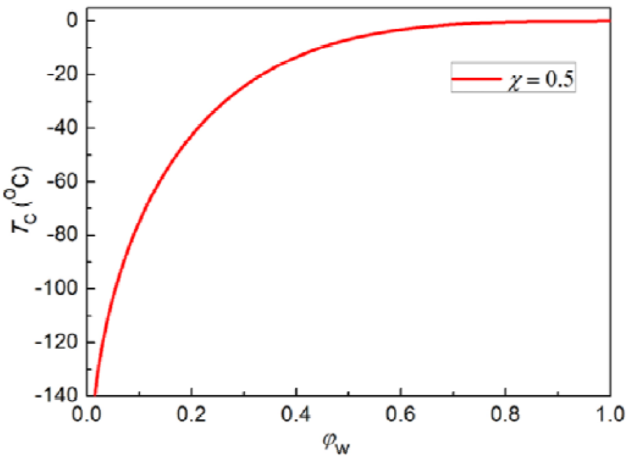

Figure S7. The freezing temperature of freezable water in the crosslinking system predicted by Flory relation for χ= 0.50.
